# Diversity of Bacteria and Yeasts Present in an Automobile Treatment System

**DOI:** 10.1007/s00248-025-02651-9

**Published:** 2025-11-20

**Authors:** Altea Villalón, Álvaro Rodríguez Alonso, Julia Carballo, Luis Alfonso Rodríguez López, María José Pérez

**Affiliations:** 1 Departamento de Bioloxía Funcional e Ciencias da Saúde (ByCIAMA group), Facultade de Ciencias, Universidade de Vigo, Ourense, 32004 Spain; 2 Instituto de Agroecoloxía e Alimentación (IAA), Universidade de Vigo-Campus Auga, Ourense, 32004 Spain

**Keywords:** Biofilm, Corrosion, Contamination, Microbial, Diversity

## Abstract

The formation of biofilms in industrial environments poses a significant challenge because of their ability to degrade materials, contaminate products, and harbour pathogenic microorganisms. In the automotive industry, surface treatment systems (STS) used to prepare car bodies can provide a favourable environment for microbial development, driven by the presence of water, organic matter, and variable physicochemical conditions. In this context, the microbial diversity present in the different STS baths of an automotive plant, as well as in the process water, was analysed. Through culture-based methods and molecular analysis, 33 bacterial and 6 yeast species were identified. The results revealed a constant presence of bacteria at all sampling points, whereas yeasts were detected less frequently and in more localized areas (Industrial and Dechromatized Water, E2, Conversion stage, E4 and Passivation stage). This study underscores the importance to enhance cleaning and disinfection protocols in STS, as high bacterial counts persisted even after rinsing stages, in order to prevent economic losses, product degradation and health risks. Furthermore, it highlights the potential use of certain microorganisms in biotechnology and bioremediation applications.

## Introduction

The deterioration of materials, such as iron and steel, is a global problem with profound social and economic consequences. This phenomenon, known as corrosion, causes losses equivalent to 3% of the world’s gross domestic product (GDP) due to the destruction of structures and the cost of their replacement [1]. Moreover, environmental regulations have increasingly restricted the use of chemical products to combat corrosion, highlighting the need to develop new sustainable and environmentally friendly control strategies [2–4].

Different products are used in each industrial process to improve the quality of the final product and prevent corrosion. Among them are metalworking fluids (MWFs), which are used as coolants and lubricants. MWFs are complex mixtures of chemicals based on oils, water, and various anticorrosion, antifoaming, and antibacterial additives [5]. These fluids become nutrient sources due to the broad variety of additives they contain, leading to unwanted microbial colonization and growth as well as physical and chemical contamination [6].

Microorganisms that grow in MWFs can adhere to different surfaces through the production of extracellular polymeric substances that promote and aid in the attachment process, forming complex structures known as biofilms [7]. Biofilms are consortia of microorganisms living within an extracellular polymeric matrix, which provides them with a protective mechanism to survive and develop under hostile conditions [8]. They have been observed in diverse settings colonizing surfaces such as catheters [9], poultry samples [10], clinical samples [11] and the automotive industry [12]. In metallic industrial environments, biofilm formation is frequently associated with genera such a *Pseudomonas*,* Bacillus* and *Acinetobacter*, which exhibit strong adhesion capabilities and metabolic versatility that facilitate their persistence on metal surface [5].

The adhesion of biofilms to metallic surfaces can alter the physical and chemical properties of materials, making them susceptible to corrosion [13], one of the biggest threats in the industry. This type of corrosion caused by microorganisms is known as biocorrosion or microbiologically influenced corrosion (MIC) [14, 15]. In addition to damaging materials and causing their deterioration, these microorganisms can lead to industrial issues, such as biofouling or even affect the quality of the final product [16].

Many microorganisms, including sulphate-reducing bacteria, iron-reducing bacteria, acid-producing bacteria and fungi, can cause the loss of lubricating and anticorrosive properties, leading to the need for frequent fluid replacement, resulting in higher costs and, therefore, economic losses [6, 17]. In general, quantifying the cost of corrosion is a complex task, but it becomes even more challenging when microorganisms are involved [18]. In addition to economic loss, biofilm formation can trigger serious health issues, as they can contain pathogenic microorganisms [19].

The automotive industry is not exempt from these problems. To remove as many adhered particles as possible and prevent them from passing to the next process, car bodies undergo a surface treatment system (STS). In this process, a protective layer is applied through a spraying system using chemicals and water to prepare the metal sheet for the next treatment layer, cataphoresis, to adhere [20]. The effectiveness of the STS is crucial, as it ensures paint quality, prolongs vehicle durability and reduces economic losses associated with production defects.

**Fig. 1:**
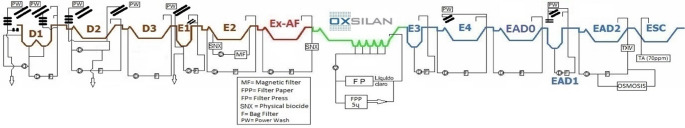
Diagram of the surface treatment system (STS): Degreasing (D1, D2, D3); Rinsing (E1-E4); Conversion (ExAfinado, OxSilan); Passivation (EAD0-EAD2); and Drain Tank (ESC)

The STS described by Núñez [20] consists of six stages comprising 13 baths: Degreasing (D1, D2, D3), Rinsing (E1, E2), Conversion (ExAfinado and OxSilan), Rinsing (E3, E4), Passivation (EAD0, EAD1, EAD2), and Drain Tank (Fig. 1).

The first stage (degreasing) removes particles, oils, and greases from the car bodies. Alkaline products are used in this process to saponify the fats, along with surfactants that physically detach the grease from the metal sheets, preventing redeposition on the metal. This phase is conducted at temperatures between 53 and 62 °C, with a pH ranging from 9.8 to 12.8.

In the next phase, Rinsing, the car bodies are washed with industrial water (E1 and E2) and deionized water (E3 and E4) to remove residues from the previously applied products. The pH of the water in Rinses 1 and 2 varies between 6.5 and 11.8, whereas it fluctuates between 4.0 and 9.0 in Rinses 3 and 4.

The Conversion stage, which includes the ExAfinado and OxSilan baths, involves immersing the car bodies in a solution containing silanes, zirconium hydroxides, and other metals to form a protective layer on the sheet surface. Because the entire system is interconnected, the ambient temperature in these phases ranges between 20 and 45 °C. The pH of the ExAfinado bath ranged between 5.0 and 9.5, while that of the OxSilan ranged from 4.3 to 5.1.

In the Passivation stage (EAD0, EAD1, and EAD2), a protective film is formed to seal the conversion layer, thereby preventing corrosion. This stage consists of deionized water with specific additives and high pH and temperature.

In the Draining stage, excess liquid is removed from the car bodies to prevent it from transferring to the cataphoresis phase. Finally, in the Cataphoresis stage, the car body is submerged in paint, and through an electric field, charged particles (paint) move toward the surface of the piece (cathode), while an auxiliary electrode (anode) completes the circuit [20].

During the production process, the automotive industry uses natural sources, such as tap water, which can act as a source of contamination. MWF contamination is common due to the transfer of fluids from one tank to another. Water systems, especially cooling systems, provide an ideal environment for microbial growth as they maintain temperatures between 25 °C and 37 °C and a pH of approximately 7. Microorganisms enter these systems from the atmosphere and water source, along with organic matter and nutrients, which promote microbial proliferation [21].

Contamination in an automobile production plant can originate from multiple sources, such as workers’ hands, sweat or saliva, the atmosphere, or even residues from the previous fluid [22]. However, the main contributor to hazardous waste is the painting process [23, 24], where the microorganisms present can metabolize organic contaminants and contribute to waste generation [25].

Understanding the microbial communities in biofilms that colonize products and materials, as well as determining the problems they may cause in industrial environments is crucial for their responsible and effective elimination. Despite the extensive information available on the structure and ecology of biofilms in different environments, a deeper approach is still needed [26].

Therefore, along with the use of molecular techniques, strategies could be developed to comprehensively understand and evaluate the microbial contamination problem. Additionally, identifying the microbial populations that compose these fluids could help develop bioremediation inocula, that is, microbial consortia specifically selected to eliminate residual fluids [5].

The objective of this study was to analyse the presence of microorganisms in different process baths and the waters used to implement antimicrobial strategies in the automotive industry to prevent both microbial contamination and associated economic losses.

## Materials and Methods

### Description of Sampling Areas

In the automobile manufacturing process, car bodies undergo a sequence of surface preparation steps designed to remove contaminants, improve paint adhesion and protect the metal from deterioration. These stages involve the application of process fluids with specific physicochemical properties. Although the exact industrial formulations are proprietary and not publicly disclosed, they are based on combinations of water, corrosion inhibitors and surfactant agents. This study specifically focused on the STS and the three types of process water involved: industrial, demineralized and dichromate.

Samples were collected in sterile 500 mL bottles from the liquid in the STS baths (Fig. 1), including Degreasing (D1, D2), ExAfinado, OxSilan, Rinse (E4), Passivation (EAD0, EAD2), and Draining, as well as from the water supplying the system (industrial, demineralized and dichromate). Sampling was performed under aseptic conditions, avoiding contact with surrounding surfaces and using sterile gloves during collection. The bottles were transported to the laboratory and stored at 3 °C until analysis.

Additionally, using sterile sponges and wipes, samples were taken from various surfaces throughout the process including: Rinse (E2, E4), ExAfinado, OxSilan, the OxSilan Stock Tank, the OxSilan sewer overflow, and Passivation (EAD0, EAD2).

Finally, sterile swabs were used to collect samples from the surfaces of the filters in: Rinse (E2, E4), ExAfinado, and OxSilan (press filter and blanket filter).

### Cultivation and Isolation Conditions

Liquid samples were serially diluted in 9 mL sterile 0.9% NaCl tubes, performing three serial dilutions for each sample. Aliquots of 0.1 mL from each dilution were spread onto plates with Tryptic Soy Agar (TSA) (Condalab) supplemented with cycloheximide (0.01% w/v) to isolate bacteria, and Sabouraud Agar (Sharlab) with chloramphenicol (0.05% w/v) to isolate fungi. The plates were incubated for 48 h at a compromise temperature of 37 °C to favour the growth of a wide range of mesophilic microorganisms. After incubation, colony-forming units (CFU/mL) were counted to determine the concentration of microorganisms present in the baths, and representative colonies were subsequently replated on fresh agar to ensure purity prior to identification.

Sponges and swabs from the surface samples were immersed in 100 mL and 9 mL, respectively, of Peptone Water and incubated at 37 °C for 24 h. Subsequently, two dilutions were prepared and processed as described above.

### Microorganism Identification

Phenotypic characterization of the colonies was performed based on shape, surface, colour, and size to determine the number of different phenotypes present in the STS system and the three types of water. Five colonies of each phenotype were arbitrarily selected and streaked onto new culture plates with both TSA and Sabouraud Agar and incubated at 37 °C for 48 h to obtain pure cultures of each selected microorganism.

Following the phenotypic characterization, the isolates were subjected to genotypic identification. For this purpose, a colony from a fresh culture was selected, and its DNA was extracted and purified using the *Bacterial & Yeast Genomic DNA Purification Kit* (EurX), according to the manufacturer’s instructions. The extracted DNA was stored at − 20 °C until further use.

For bacteria, a fragment of approximately 1500 pb of the 16 S rRNA was amplified using the primers 27 F and 1492R, with the following sequences:

- 27F: AGA GTT TGA TCM TGG CTC AG
- 1492R: GGT TAC CTT GTT ACG ACTT

For yeasts, the D1/D2 region of the 26 S rRNA gene was amplified using the NL1 and NL4 primers, with the following sequences:

- NL1: GCA TAT CAA TAA GCG GAG GAA AAG
- NL4: GGT CCG TGT TTC AAG ACGG

PCR reactions were performed in a *ProFlex PCR System* thermocycler (Applied Biosystems). Each reaction (50 µL) was prepared using the *DreamTaq DNA Polymerase* kit (Thermo Scientific), with 20 pmol of each primer and 1 µL of DNA template. The amplification program consisted of 35 cycles under the following conditions:

- denaturation at 95 °C for 30 s,
- annealing at 55 °C for 30 s,
- extension at 72 °C for 1 min.

A final extension step was performed at 72 °C for 10 min.

PCR products were analyzed by electrophoresis on 1% (w/v) agarose gels prepared in 1× TBE buffer (Sigma, St. Louis, Missouri, USA).

The amplified fragments were purified using the *NucleoSpin Gel and PCR Clean-up* kit (Macherey-Nagel) and subsequently sequenced with the *BigDye Terminator v3.1 Cycle Sequencing* Kit (Applied Biosystems) on an ABI 3500 capillary automated sequencer (Applied Biosystems), according to the manufacturer’s instructions.

The resulting sequences were assembled into contigs using the BioEdit Biological Sequence Alignment Editor. For taxonomic identification, sequences were compared against the GenBank database of the National Center for Biotechnology Information (NCBI; http://www.ncbi.nlm.nih.gov) using the BLAST tool (http://www.ncbi.nlm.nih.gov/BLAST).

### Diversity and Evenness Analysis

To evaluate the relative distribution of microorganisms in each sample, the equitability of species (Evenness, E) was calculated. First, the proportion (p_i_) of each species was determined based on the number of colonies isolated relative to the total number of colonies in the sample. The Shannon diversity index

$$H'=\Sigma p_i\cdot\ln\left(p_i\right))$$

was then calculated, and Evenness was obtained by normalizing H’ by the total number of species present (S) using the formula $\:E=H'/\:ln\left(S\right)$.

Calculations were performed using Microsoft Excel, applying standard formulas for proportions and natural logarithms. E values range from 0 to 1, where 0 indicates dominance by a few species and 1 reflects an even distribution of all species.

## Results and Discussion

### Microorganism Count

The analysis of the samples collected from the STS baths, based on a single sampling event, allowed the determination of the bacterial and yeast concentrations in the system. Bacterial growth was observed at all sampled points, suggesting a constant presence of microorganisms throughout the process.

However, the yeast distribution was irregular and limited to certain baths. In some of these baths, the concentration was very low or undetectable, which may suggest that yeasts do not proliferate uniformly throughout the system. The exact composition of each bath is unknown; however, since body cars sequentially pass through the baths, their composition likely changes over time. Therefore, the observed differences in yeast distribution could be due to variations in environmental conditions or changes in the chemical composition of the liquid in each bath.

Since the system employs a cascading water circuit, it not only promotes water conservation but might also facilitates cross-contamination throughout the process. Although this approach is ecologically beneficial the transfer of water between different baths could enable the spread of microorganisms.

**Fig. 2:**
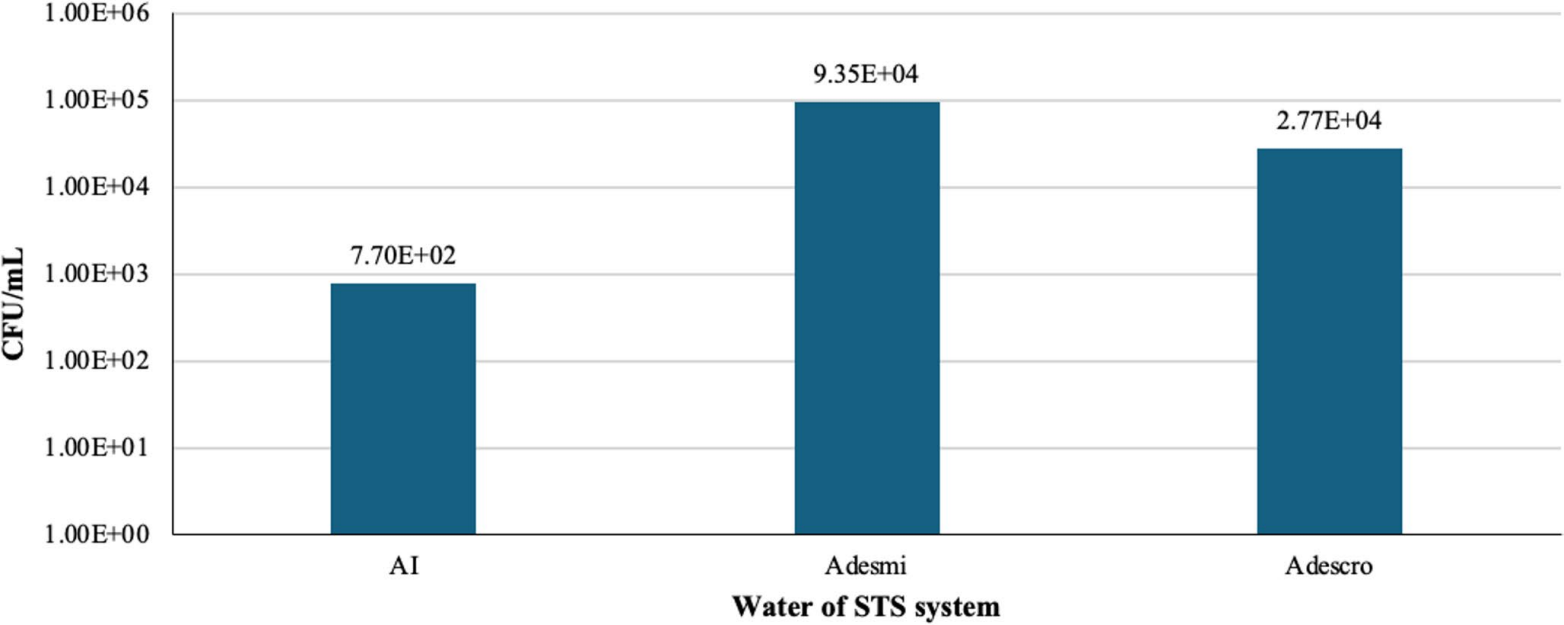
Bacterial counts (CFU/mL) in the water of STS system. From left to right, the categories include Industrial Water (AI), Demineralized Water (Adesmi) and Dechromatized Water (Adescro)

The results indicate differences in the bacterial load between the different baths (Fig. 2). Samples from demineralized water (Adesmi) and dechromatized water (Adescro) showed high bacterial counts, with Adesmi reaching the highest value (9.35 × 10^4^ CFU/mL). This could suggest that the microbiological quality of the process water may contribute to the contamination downstream of the baths supplied by sources such as ExAfinado, OxSilan, EAD0, and EAD2.

**Fig. 3:**
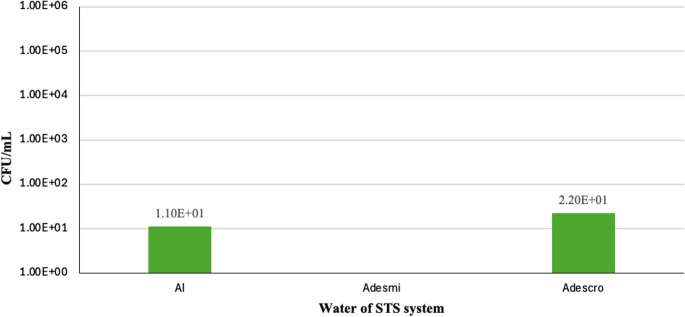
Yeast counts (CFU/mL) in the water of STS system. From left to right: Industrial water (AI), Demineralized water (Adesmi), and Dechromatized water (Adescro))

Unlike bacteria, the waters used did not show a high concentration of yeasts, which might indicate that they are not the primary source of yeast contamination, as appears to be the case of bacteria (Fig. 3).

**Fig. 4:**
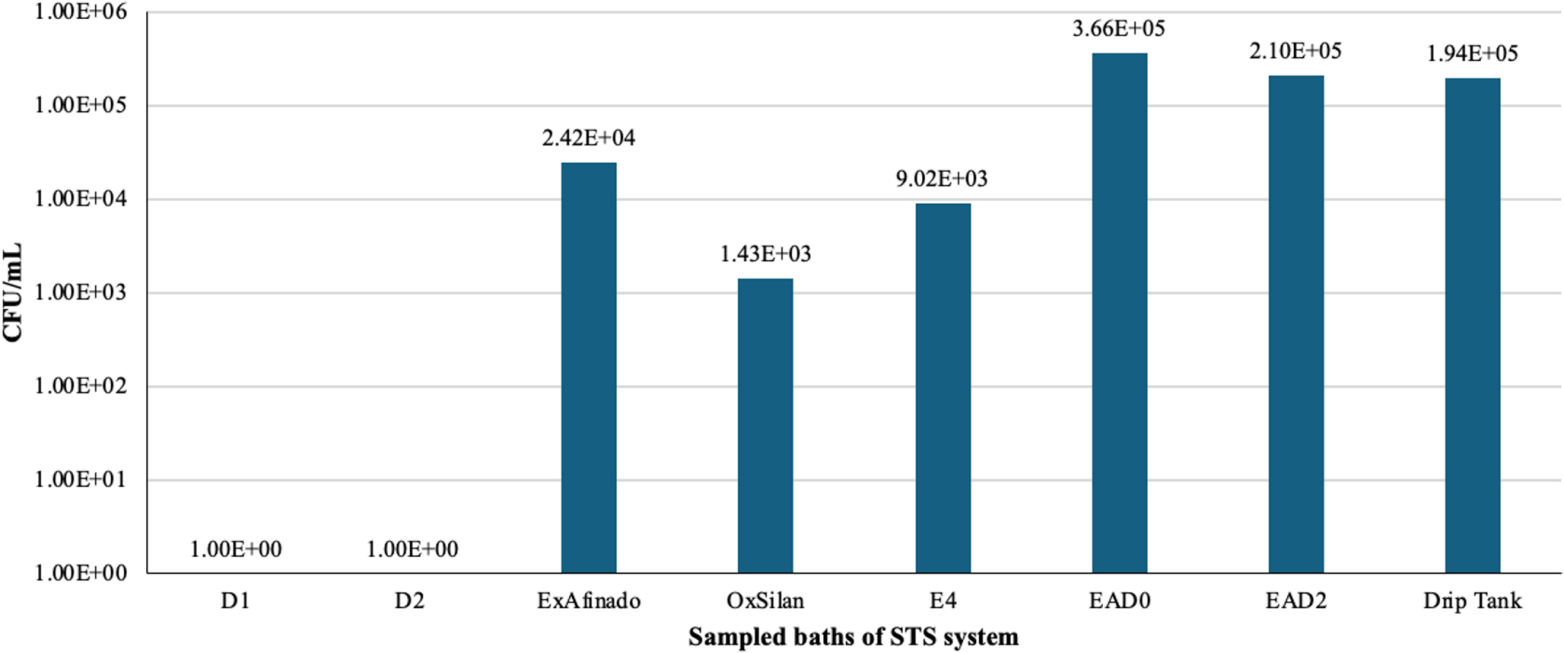
Bacterial counts (CFU/mL) in the liquid from the sampled baths (From left to right: Degreasing (D1 and D2), ExAfinado, OxSilan, Rinse (E4), Passivation (EAD0, EAD2), Drip tank)

Figure 4 illustrates that the Passivation baths (EAD0 and EAD2) had the highest bacterial concentrations, with 3.66 × 10^5^ CFU/mL in EAD0 and 2.10 × 10^5^ CFU/mL in EAD2. This could suggest that these baths are particularly prone to bacterial proliferation. The specific conditions in these baths, such as high pH and elevated temperatures, may facilitate bacterial growth, as bacteria generally have faster growth rates compared to yeasts [27]. Furthermore, these baths are supplied with demineralized and dichromatized water sources, which already showed high bacterial concentrations, suggesting that these sources could be the primary contributors to contamination.

In contrast, the OxSilan bath exhibited the lowest bacterial concentration (1.43 × 10^3^ CFU/mL). This could be attributed to its acidic pH (≈ 4,3–5,1), which is unfavourable for most bacteria. Similarly, Rinse E4 displayed a low bacterial concentration (9.02 × 10^3^ CFU/mL), possibly due to the low bacterial load carried over from the OxSilan bath.

However, after Rinse E4, an increase in bacterial concentration was observed in the Passivation stage (EAD0 and EAD2). This may suggest that the conditions in these baths are favourable for bacterial growth, despite the previous rinsing step. Although these baths mainly consist of deionized water with specific additives and elevated pH and temperature, residual organic matter or trace compounds from previous stages could potential carbon sources supporting microbial development.

**Fig. 5:**
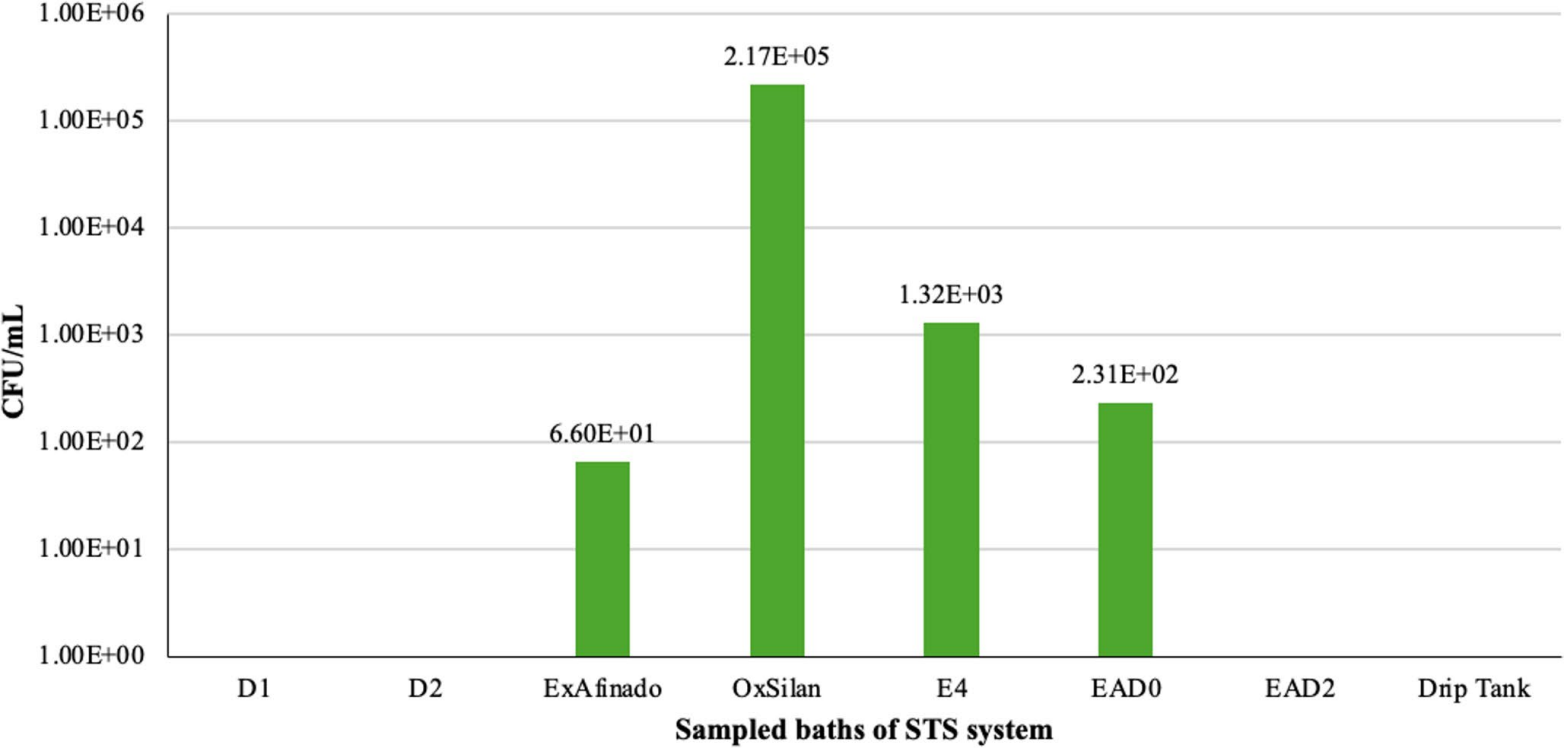
Yeast counts (CFU/mL) in the liquid from the sampled baths where presence was detected (From left to right: Degreasing (D1 and D2), ExAfinado, OxSilan, Rinse (E4), Passivation (EAD0, EAD2), Drip tank)

Regarding yeasts in the liquids, the OxSilan bath showed an interesting behaviour, with a high concentration (2.17 × 10^5^ CFU/mL) (Fig. 5). Unlike bacteria, yeasts were not detected in significant amounts in the waters used, which could indicate that certain characteristics of the OxSilan bath, particularly its low pH (4,3–5,1), might favour their proliferation. However, further investigation would be needed to confirm this hypothesis.

The concentration of microorganisms in the baths and process water varies significantly due to the specific conditions of each bath and the quality of the water used. The high concentrations of bacteria in the Passivation baths (EAD0 and EAD2) and in the process waters suggest that contamination may be introduced through the water supply, highlighting the importance of microbiological quality control in the water used throughout the process.

**Fig. 6:**
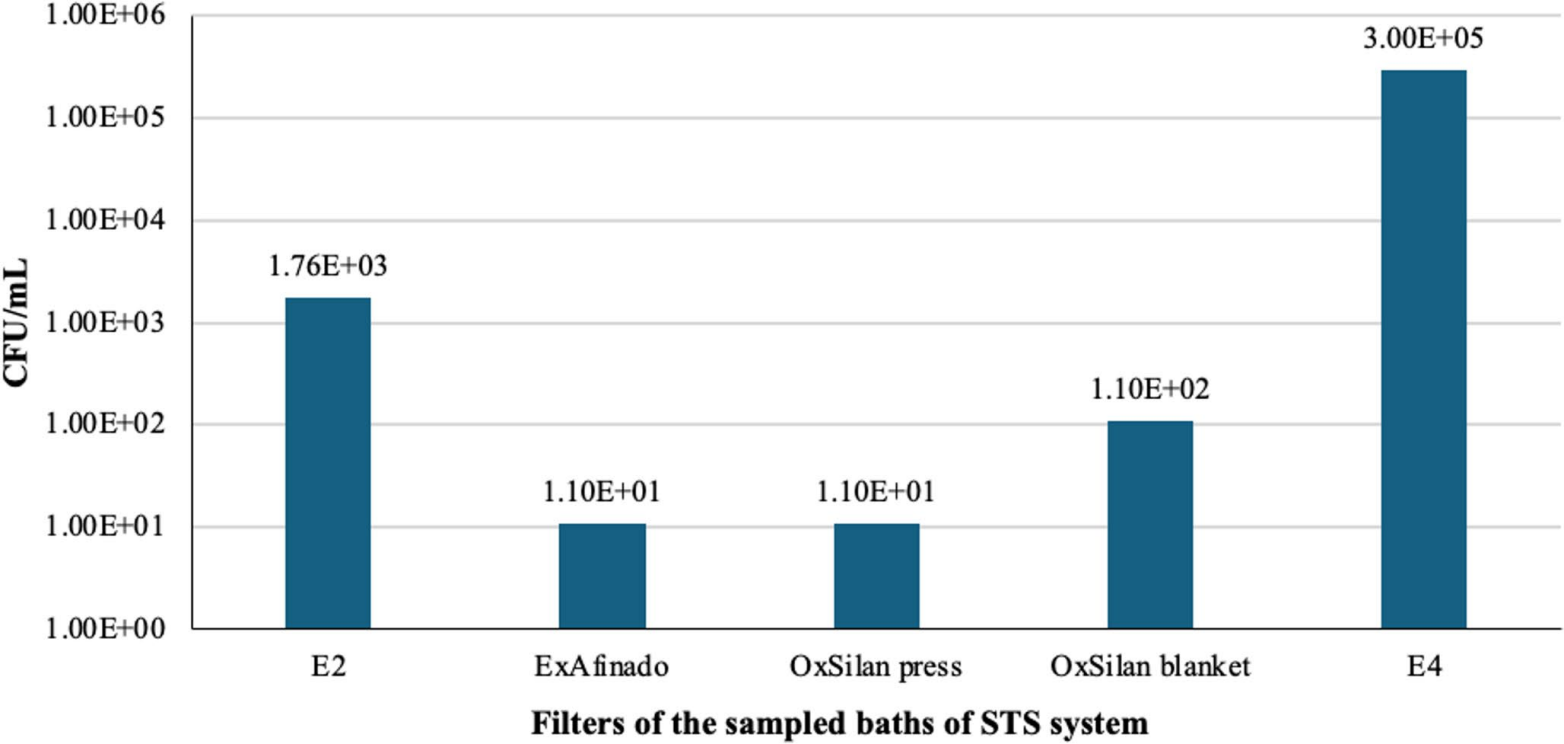
Bacterial counts (CFU/mL) in the filters of the sampled baths (From left to right: Rinse (E2), ExAfinado, OxSilan press filter, OxSilan manta filter, Rinse (E4))

In the filters, a high concentration of bacteria was observed, especially in filter E4 (> 3 × 10^5^ CFU/mL) (Fig. 6), which could result from the carryover and accumulation of microorganisms from other baths. This finding underscores the importance of monitoring and cleaning the filters, as they can serve as reservoirs for microorganisms, facilitating their spread to other areas of the process.

**Fig. 7:**
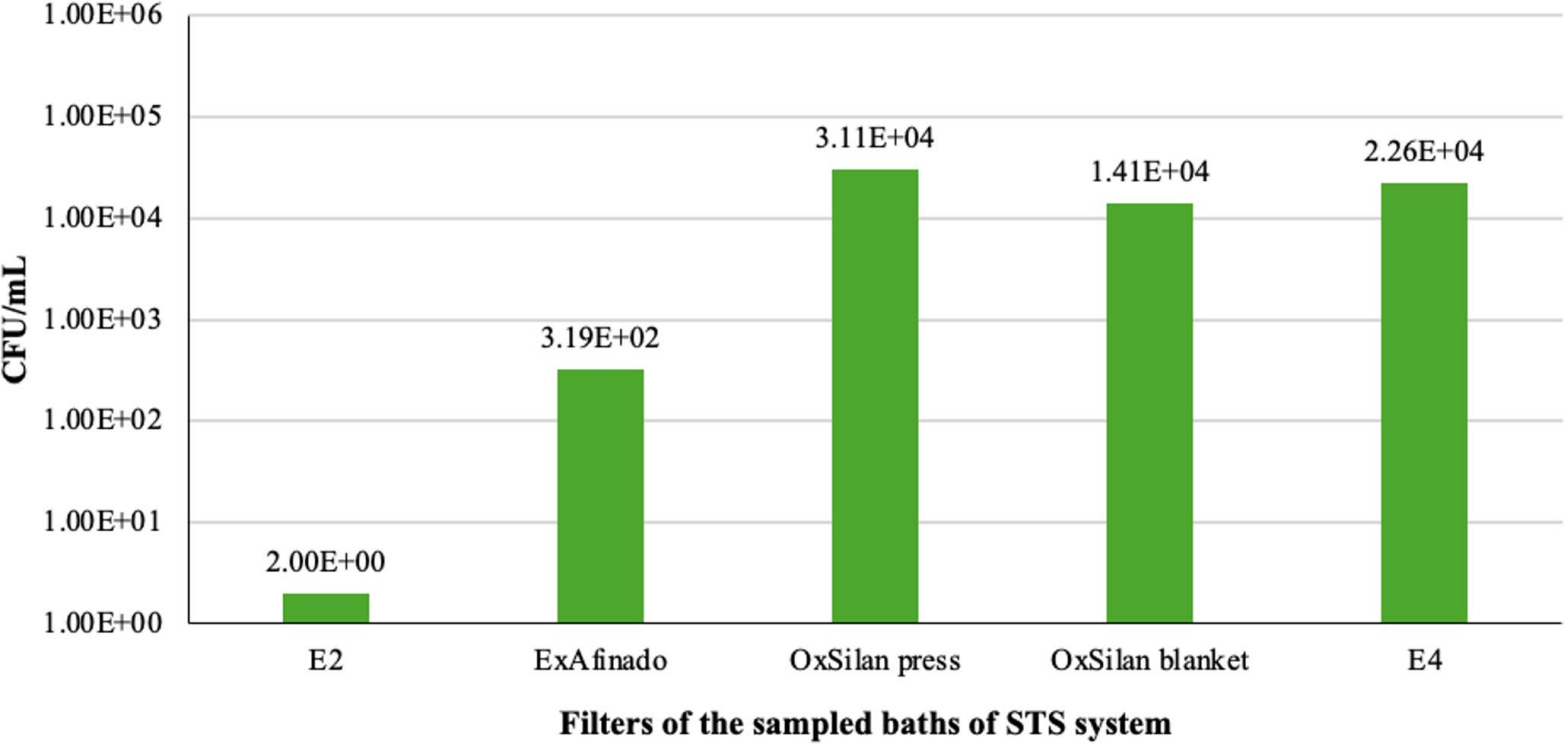
Yeast counts (CFU/mL) in the filters of the sampled baths (From left to right: Rinse (E2), ExAfinado, OxSilan press filter, OxSilan manta filter, Rinse (E4))

On the other hand, yeasts were primarily concentrated in the OxSilan press filter (3.11 × 10^4^ CFU/mL) and manta filter (1.41 × 10^4^ CFU/mL), suggesting that the conditions in this bath might promote their accumulation (Fig. 7). The high yeast concentration in the press filter corresponds to the elevated concentration in the bath liquid, which may indicate that the specific conditions of OxSilan are particularly favourable for their growth.

**Fig. 8:**
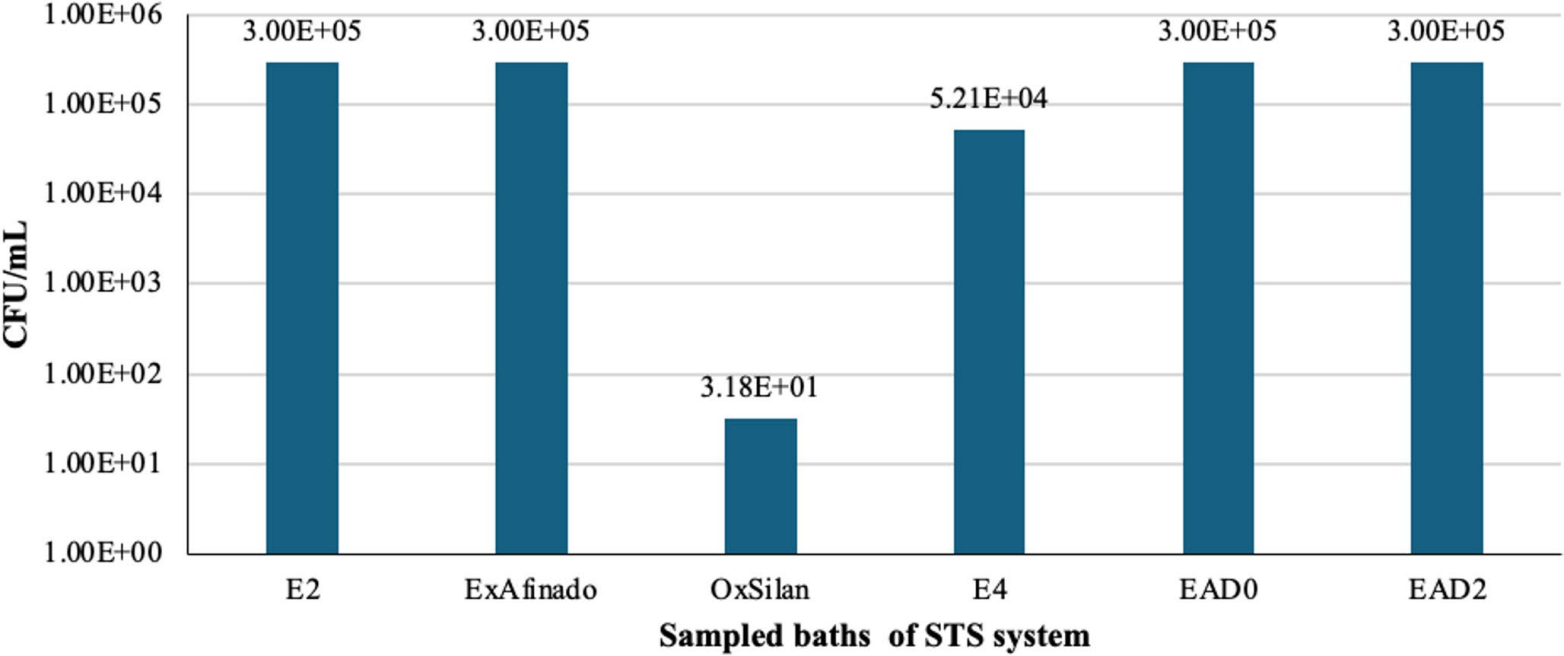
Bacterial counts (CFU/mL) on the surfaces of the sampled baths (From left to right: Rinse (E2), ExAfinado, OxSilan, Rinse (E4), Passivation (EAD0, EAD2))

On the surfaces of the baths, as shown in Fig. 8, significant concentrations of bacteria were observed on the surfaces of E2, ExAfinado, EAD0 and EAD2 (> 3 × 10^5^ CFU/mL), possibly indicating the accumulation of microorganisms due to the specific conditions of these baths (water quality, bath pH, temperature, and available organic matter). These conditions may favour the formation of biofilms, which are often difficult to eliminate with standard cleaning procedures and may act as recurring sources of contamination.

Regarding yeast, their presence on the surfaces of the OxSilan (1.66 × 10 CFU/mL) and E4 (2.53 × 10 CFU/mL) baths was lower compared to their concentration in the bath liquid. This may indicate that, in this case, yeasts are predominantly planktonic rather than adhering to the surfaces.

**Fig. 9:**
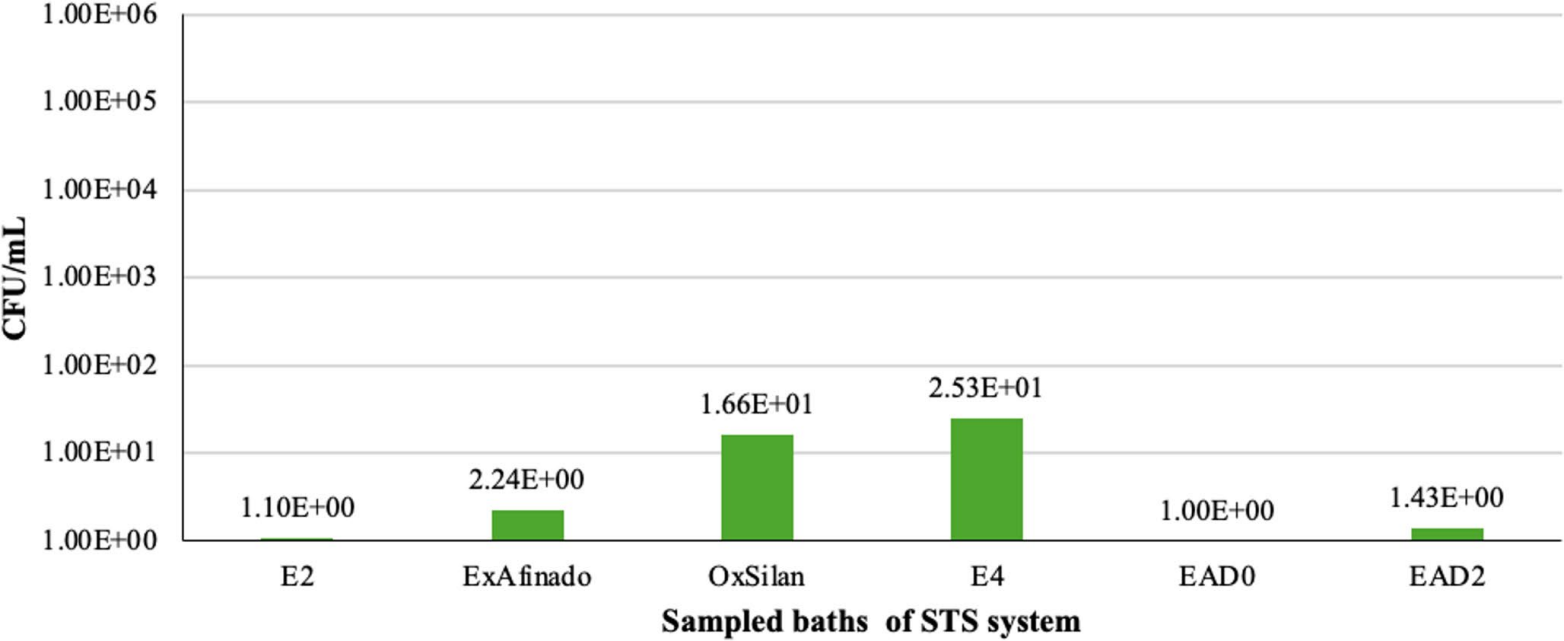
Yeast counts (CFU/mL) on the surfaces of the sampled baths (From left to right: Rinse (E2), ExAfinado, OxSilan, Rinse (E4), Passivation (EAD0, EAD2))

It is important to consider that the baths receive contributions of grease, minerals, and organic matter from various sources (car bodies, guides, and operators), which could create a favourable environment for biofilm formation. Additionally, hard-to-clean areas may become reservoirs for biofilms, allowing contamination to persist despite cleaning procedures. The products used in the baths might also act as selective nutrients, promoting the growth of certain microorganisms and contributing to the increased microbial load in the system (Fig. 9).

### Microorganism Identification

Molecular analysis identified 33 bacterial species (Table 1) and 6 yeast species (Table 2) in the STS system baths. Due to differences in the pH, temperature, and chemical composition of the baths, it was observed that certain species predominated under specific conditions, suggesting that their removal could be achieved through the modification of these parameters or the application of targeted antimicrobial strategies.

**Table 1:** Bacterial diversity found in the STS system baths

Class	Specie	Isolated from	Evenness (E)	%Homology
Actinobacteria	*Dietzia aurantiaca*	Adesmi, E2, OxS, E4, EAD0, EAD2, ESC	0,377	> 99
	*Haematomicrobium sanguinis*	OxS, E4	0,307	> 99
	*Leifsonia shinshuensis*	ADescro, E4, EAD0, EAD2	0,284	> 99
	*Microbacterium paraoxydans*	OxS, E4	0,252	> 99
	*Micrococcus luteus*	ExAF, OxS	0,250	> 99
	*Nocardia farcinica*	E2	0,235	> 99
	*Tsukamurella inchonensis*	ExAF, OxS	0,001	> 99
Alphaproteobacteria	*Roseomonas mucosa*	OxS	0,132	> 99
Bacilli	*Bacillus altitudinis*	E2, OxS, E4	0,642	> 99
	*Bacillus cereus*	AI, E2, EXaF, OxS, E4, EAD2, ESC	0,611	> 99
	*Bacillus licheniformis*	E4	0,600	> 99
	*Bacillus subtilis*	E4, EAD0	0,594	> 99
	*Cytobacillus horneckiae*	Adesmi	0,389	> 99
	*Exiguobacterium profundum*	E2	0,335	> 99
	*Jeotgalicoccus aerolatus*	EAD0, EAD2	0,286	> 99
	*Lysinibacillus macroides*	AI, OxS	0,253	> 99
	*Paenibacillus taichungensis*	OxS	0,232	> 99
	*Priestia megaterium*	OxS, ESC	0,231	> 99
	*Shouchella rhizosphaerae*	E4	0,131	> 99
	*Staphylococcus capitis*	OxS	0,114	> 99
	*Staphylococcus epidermidis*	AI	0,093	> 99
	*Staphylococcus saprophyticus*	AI, EAD0, EAD2, ESC	0,092	> 99
	*Staphylococcus pseudoxylosus*	EAD2	0,025	> 99
Betaproteobacteria	*Alicycliphilus denitrificans*	ExAf	0,670	> 99
	*Cupriavidus malaysiensis*	ADescro, E4	0,529	> 99
	*Cupriavidus metallidurans*	ADescro, ExAF, OxS, E4, EAD0, EAD2, ESC	0,492	> 99
Gammaproteobacteria	*Acinetobacter junii*	ExAf	0,734	> 99
	*Acinetobacter modestus*	EAD2	0,700	> 99
	*Alishewanella jeotgali*	E2	0,670	> 99
	*Pseudomonas alcaligenes*	E2	0,222	> 99
	*Pseudomonas guguanensis*	ExAf	0,161	> 99
	*Stenotrophomonas maltophilia*	ExAf	0,006	> 99
	*Thermomonas haemolytica*	ExAf	0,002	99

* AI: Industrial Water; Adesmi: Demineralized Water; Adescro: Dechromatized Water. ExAf: ExAfinado; OxS: OxSilan; E2-E4: Ringe; EAD0-EAD2: Passivation; ESC: Drip tank

**Table 2:** Yeast diversity found in the STS system baths

Class	Specie	Isolated from	Evenness (E)	%Homology
Microbotryomycetes	*Rhodotorula toruloides*	OxS	0,029	> 99
Saccharomycetes	*Candida orthopsilosis*	OxS	0,641	> 99
	*Candida palmioleophila*	AI, ADescro, E2, ExAf, OxS, E4, EAD0	0,435	> 99
	*Candida parapsilosis*	E2, ExAf, OxS, E4, EAD2	0,232	> 99
Taphrinomycetes	*Taphrina coerulescens*	EAD0	0,024	> 99
Tremellomycetes	*Tricchosporiella flavicans*	EAD2	0,019	> 99

* AI: Industrial Water; Adescro: Dechromatized Water.; ExAf: ExAfinado; OxS: OxSilan; E2-E4: Ringe; EAD0-EAD2: Passivation

Among the species identified in the STS system, several are relevant not only for bioremediation and resistance to contaminants, but also for their ability to form biofilms, which may explain the microbial distribution patterns described in Sect. 3.1. In the degreasing and initial rinse baths (E1 and E2), where fats and oils were present, bacteria capable of hydrocarbon degradation were detected. *Pseudomonas alcaligenes* and *Microbacterium paraoxydans* have proven to be highly efficient in degrading these compounds, which could explain their persistence in these baths [28, 29]. Moreover, *Pseudomonas* is a well-known biofilm producer and species of genus *Microbacterium* have been reported to adhere to surfaces and participate in biofilm formation in industrial environments [30, 31]. *Thermomonas haemolytica*, recently reported by [32], also stands out in the degradation of organic pollutants, suggesting its involvement in the removal of industrial waste within the system. In addition, species of *Thermomonas sp*. have been isolated from biofilms, indicating their capacity to contribute to biofilm development [33]. The abundance of these genera in the early stages of the process supports their active role in the initial establishment of biofilms within the STS system.

In the Passivation baths, characterized by high pH and temperatures, species resistant to heavy metals were detected. *Cupriavidus metallidurans* and *Paenibacillus taichungensis* have been widely studied for their ability to survive in environments with high metal loads [34, 35], such as the STS system, where metallic salts are used in the Conversion and Passivation processes. Both genera are known to adhere to surfaces and produce biofilms, which may protect them from harsh conditions and promote their survival [36, 37]. Similarly, *Alishewanella* has shown effectiveness in the bioremediation of heavy metals such as chromium and cadmium [38], while *Lysinibacillus macroides* can degrade chlorobenzoic compounds [39] suggesting its role in the remediating of organochlorine compounds in industrial baths. Their detection in baths with extreme pH is consistent with the formation of mature biofilms that allow these microorganisms to persist under extreme physicochemical conditions [40, 41].

Other microorganisms with degradation capacity include *Leifsonia shinshuensis*, which can metabolize drugs like carbamazepine, an emerging contaminant in wastewater [42], and *Dietzia aurantiaca*, which has proven effective in the degradation of fluorotelomers, which are persistent contaminants in industrial settings [43].

On the other hand, some identified bacteria have potential in the production of industrially relevant metabolites. *P. guguanensis* has been isolated for its use in the production of eco-friendly hydrocarbon dispersants [44]. *Thermomonas haemolytica*, in addition to its degradation capacity, can produce detergents thanks to its protease enzyme [45], suggesting that its presence in rinse baths may be related to the degradation of chemical residues.

Other biotechnologically relevant species include *Tsukamurella inchonensis*, which could have applications in the treatment of metabolic diseases such as diabetes [46], and *Priestia megaterium*, which is used in the production of vitamin B12 and biopolymers [47]. Moreover, *Bacillus licheniformis* is known for its ability to produce antibiotics [48, 49] and its use in bioremediation [50], while *B. subtilis* is employed as a probiotic [51] and plant growth promoter [52]. Many *Bacillus* species are also strong biofilm formers, which may contribute to their persistence across multiple baths in the system [53].

Some Proteobacteria play key roles in the nitrogen cycle. *Alicycliphilus denitrificans* can oxidize ammonium to nitrite (nitrification) and reduce nitrate to nitrite (denitrification), contributing to the transformation of nitrogen in the STS system baths [54]. Its presence in the Passivation and Rinse baths suggests that it may be involved in the recycling of nitrogen compounds.

Among the isolated species, several are pathogenic and may pose a risk in industrial environments. *Nocardia farcinica* [55] and *B. cereus*, the cause of food poisoning [56], have been detected at several points in the system. *Staphylococcus epidermidis*, a commensal member of human skin, can cause opportunistic infections [57] and has been found on bath surfaces, suggesting possible cross-contamination in the process. Similarly, *S. saprophyticus*, associated with urinary tract infections [58], and *S. capitis*, a relevant pathogen in hospitalized babies [59], have been identified in the Passivation baths, indicating that high pH alone is not sufficient to completely inhibit their growth. Although *Micrococcus luteus* is generally not considered a pathogen, strains have been reported to cause endocarditis and bacteraemia [60]. Many of these opportunistic pathogens are also known biofilm formers, which may facilitate their persistence on industrial surfaces.

Among the genera with increasing clinical relevance, *Acinetobacter junii* and *A. modestus* have been identified in the STS system. These species have shown antibiotic resistance [61, 62], and their detection in the system suggests that they may survive under adverse conditions, such as extreme pH baths, possibly through biofilm-mediated protection mechanisms [63].

Regarding yeasts (Table 2), they have been primarily identified in the Conversion baths, where low pH conditions favour their proliferation. The *Candida* genus is one of the most common in industrial processes and has been widely reported in environments with acidic pH due to its ability to withstand extreme conditions such as high sugar concentrations and low water activity [64]. In the Conversion baths, particularly OxSilan, species such as *C. palmioleophila*,* C. parapsilosis*, and C. *orthopsilosis* were identified, suggesting that these yeasts find optimal conditions for growth in these environments [65, 66].

Yeast cells have a high capacity to adhere to abiotic surfaces, cells, and tissues, which gives them significant relevance in industrial environments. The formation of dense biofilms, with hyphae and pseudohyphae, makes their removal difficult and increases the risk of recurrent contamination in the system baths [67]. The presence of *C. palmioleophila* in the Conversion baths reinforces this issue, as this species has become a relevant pathogen in recent years with increasing resistance to antimicrobials [68, 69].

Despite their potential negative impact on the STS system, some yeasts have industrial applications of interest. *Candida* genus can produce biosurfactant with beneficial properties for industrial processes, sparking interest in their use for various biotechnological applications [70, 71]. Additionally, *Rhodotorula toruloides* was detected in high concentrations in OxSilan and is notable for its ability to accumulate intracellular lipids, making it useful for biodiesel production [72].

The presence of yeasts in the STS system, especially in the Conversion baths, suggests that low pH conditions favour their proliferation and biofilm formation. Their removal requires specific strategies, such as using antifungals compatible with the process and optimizing the bath replacement frequency.

Several of the bacterial genera identified in this study, including *Pseudomonas*, *Bacillus* and *Acinetobacter*, have previously been associated with MIC processes [5]. These microorganisms can contribute to material degradation through biofilm formation. The physicochemical characteristics of certain baths, such as high pH and temperature during the Passivation stage, may promote biofilm development and influence corrosion potential. Although direct corrosion measurements were not conducted, the detection of these microorganisms at critical stages of the STS suggests that they could contribute to corrosion phenomena within the system.

Despite these relevant observations, some methodological constraints should be considered when interpreting the data. It is important to acknowledge some limitations of this study that should be considered when interpreting the results. First, the analyses were based on culture-dependent methods, which may underestimate the total microbial diversity present in the system, particularly for fastidious or non-culturable organisms. Additionally, TSA and Sabouraud media and incubation at 37 °C were selected to reproduce the conditions of the baths, which operate at temperatures above ambient; however, this choice may have biased the isolation toward mesophilic microorganisms capable of growing under these laboratory conditions, potentially excluding psychrotolerant, thermotolerant, or slow-growing species present in the STS. Second, samples were collected at a single time point, therefore do not capture possible temporal fluctuations in microbial communities. Third, no direct corrosion measurements or surface damage analyses were conducted; consequently, the potential role of the identified microorganisms in MIC processes remains hypothetical. Future studies combining culture-independent approaches and corrosion rate measurements would provide a more comprehensive understanding of microbial dynamics and their impact on material degradation in STS.

## Conclusions

To the best of our knowledge, this study may represent one of the first descriptions of the presence of bacteria and yeasts in an automotive surface treatment system. The results obtained suggest that the composition of the different baths in the factory could influence the concentration and diversity of the present microorganisms. While some baths show high levels of bacteria, others, such as the OxSilan bath are dominated by yeast.

Variations in pH and temperature in the STS system baths could be related to the different distribution of microorganisms. In particular, the Passivation baths (EAD0 and EAD2), which have high pH and elevated temperature, seem to favour bacterial growth, as the highest concentrations have been recorded there. In contrast, the OxSilan bath, with a more acidic pH, had lower bacterial concentrations, suggesting that such conditions may not be conducive to their development. However, this same bath exhibited a high yeast concentration, which may indicate that its chemical composition and environmental conditions could be more favourable for these microorganisms.

Regarding the Rinse baths, the data indicate that they do not achieve a complete reduction in microbial load unless their function is to eliminate contaminants. This may explain the presence of bacteria in subsequent stages of the process, such as in the Passivation baths. Furthermore, the filters, particularly at point E4, have been found to accumulate significant amounts of bacteria and yeast, raising the possibility that they act as microbial reservoirs and contribute to the spread of microorganisms throughout the system.

The microbial counts obtained reinforce the idea that cleaning and disinfection protocols should be reviewed, with particular attention to the choice and concentration of the products used. It is worth noting that the gradual replacement of traditional treatments by more environmentally friendly technologies—such as the OxSilan process—may have created more favourable conditions for certain microorganisms, especially when combined with increased production demands.

Since the presence of bacteria and yeast could be associated with product deterioration, economic losses, and even—outside the industrial environment—with health risks, it would be advisable to pay greater attention to their potential adhesion to equipment surfaces.

Detection of species with pathogenic and biotechnological potential opens new lines of research. These could focus on identifying more effective strategies to reduce microbial contamination and harnessing certain microorganisms for beneficial purposes, such as industrial waste bioremediation.

In upcoming trials, the possibility of testing different concentrations and combinations of disinfectants authorized for use in the plant is being considered to evaluate their effectiveness. Improving current protocols and designing more effective strategies could make a significant contribution to optimizing product quality and overall industrial process efficiency.

## Data Availability

No datasets were generated or analysed during the current study.

## References

[1] Emerson D (2018) The role of iron-oxidizing bacteria in biocorrosion: a review. Biofouling 34(9):989–1000. 10.1080/08927014.2018.1526281 30642207 10.1080/08927014.2018.1526281

[2] Bender R, Féron D, Mills D, Ritter S, Bäßler R, Bettge D, Zheludkevich M (2022) Corrosion challenges towards a sustainable society. Mater Corros 73(11):1730–1751. 10.1002/maco.202213140

[3] Aljibori HS, Alamiery A, Kadhum AAH (2023) Advances in corrosion protection coatings: A comprehensive review. Int J Corros Scale Inhib 12(4):1476–1520. 10.17675/2305-6894-2023-12-4-6

[4] Aljibori H, Al-Amiery A, Isahak WNR (2024) Advancements in corrosion prevention techniques. J Bio-and Tribo-Corrosion 10(4):78. 10.1007/s40735-024-00882-w

[5] Van der Gast CJ, Whiteley AS, Lilley AK, Knowles CJ, Thompson IP (2003) Bacterial community structure and function in a metal-working fluid. Environ Microbiol 5(6):453–461. 10.1046/j.1462-2920.2003.00428.x 12755712 10.1046/j.1462-2920.2003.00428.x

[6] Saha R, Donofrio RS (2012) The microbiology of metalworking fluids. Appl Microbiol Biotechnol 94:1119–1130. 10.1007/s00253-012-4055-7 22543351 10.1007/s00253-012-4055-7

[7] Salgar-Chaparro SJ, Lepkova K, Pojtanabuntoeng T, Darwin A, Machuca LL (2020) Nutrient level determines biofilm characteristics and subsequent impact on microbial corrosion and biocide effectiveness. Appl Environ Microbiol 86(7):e02885–e02819. 10.1128/AEM.02885-19 31980429 10.1128/AEM.02885-19 PMC7082584

[8] Galie S, García-Gutiérrez C, Miguélez EM, Villar CJ, Lombó F (2018) Biofilms in the food industry: health aspects and control methods. Front Microbiol 9:898. 10.3389/fmicb.2018.00898 29867809 10.3389/fmicb.2018.00898 PMC5949339

[9] Niveditha S, Pramodhini S, Umadevi S, Kumar S, Stephen S (2012) The isolation and the biofilm formation of uropathogens in the patients with catheter associated urinary tract infections (UTIs). J Clin Diagn Research: JCDR 6(9):1478. 10.7860/JCDR/2012/4367.2537 23285434 10.7860/JCDR/2012/4367.2537 PMC3527774

[10] Merino L, Procura F, Trejo FM, Bueno DJ, Golowczyc MA (2019) Biofilm formation by *Salmonella sp*. in the poultry industry: detection, control and eradication strategies. Food Res Int 119:530–540. 10.1016/j.foodres.2017.11.024 30884686 10.1016/j.foodres.2017.11.024

[11] Nirwati H, Sinanjung K, Fahrunissa F, Wijaya F, Napitupulu S, Hati VP, Hakim MS, Meliala A, Aman AT, Nuryastuti T (2019), December Biofilm formation and antibiotic resistance of *Klebsiella pneumoniae* isolated from clinical samples in a tertiary care hospital, Klaten, Indonesia. In *BMC proceedings* (Vol. 13, No. 11, pp. 1–8). BioMed Central. 10.1186/s12919-019-0176-7 10.1186/s12919-019-0176-7 PMC6913045 31890013

[12] Radojević ID, Grujić SM, Ranković BR, Čomić LR, Ostojić AM (2019) Single-species biofilms from autochthonous microorganisms: biotechnological potential in automotive wastewater treatment. Int J Environ Sci Technol 16:6189–6198. 10.1007/s13762-019-02265-y

[13] Javed MA, Stoddart PR, Palombo EA, McArthur SL, Wade SA (2014) Inhibition or acceleration: bacterial test media can determine the course of microbiologically influenced corrosion. Corros Sci 86:149–158. 10.1016/j.corsci.2014.05.003

[14] Beech IB, Sunner J (2004) Biocorrosion: towards understanding interactions between biofilms and metals. Curr Opin Biotechnol 15(3):181–186. 10.1016/j.copbio.2004.05.001 15193324 10.1016/j.copbio.2004.05.001

[15] Guo J, Yuan S, Jiang W, Lv L, Liang B, Pehkonen SO (2018) Polymers for combating biocorrosion. Front Mater 5:10. 10.3389/fmats.2018.00010

[16] Trafny EA, Lewandowski R, Kozłowska K, Zawistowska-Marciniak I, Stępińska M (2015) Microbial contamination and biofilms on machines of metal industry using metalworking fluids with or without biocides. Int Biodeterior Biodegrad 99:31–38. 10.1016/j.ibiod.2014.12.015

[17] Hou B, Li X, Ma X, Du C, Zhang D, Zheng M, Weichen X, Dongzhu L, Ma F (2017) The cost of corrosion in China. Npj Mater Degrad 1(1):4. 10.1038/s41529-017-0005-2

[18] Zhu XY, Lubeck J, Kilbane JJ (2003) Characterization of microbial communities in gas industry pipelines. Appl Environ Microbiol 69(9):5354–5363. 10.1128/AEM.69.9.5354-5363.2003 12957923 10.1128/AEM.69.9.5354-5363.2003 PMC194955

[19] Abebe GM (2020) The role of bacterial biofilm in antibiotic resistance and food contamination. Int J Microbiol. 10.1155/2020/1705814 32908520 10.1155/2020/1705814 PMC7468660

[20] Núñez M (2015) Reducción de partículas ocluidas en carrocería cataforizada en una instalación de pintura de automóviles [Tesis de doctorado] Universidad de Vigo

[21] Liu Y, Zhang W, Sileika T, Warta R, Cianciotto NP, Packman AI (2011) Disinfection of bacterial biofilms in pilot-scale cooling tower systems. Biofouling 27(4):393–402. 10.1080/08927014.2011.577525 21547755 10.1080/08927014.2011.577525 PMC4507511

[22] Van der Gast CJ, Knowles CJ, Wright MA, Thompson IP (2001) Identification and characterisation of bacterial populations of an in-use metal-working fluid by phenotypic and genotypic methodology. Int Biodeterior Biodegrad 47(2):113–123. 10.1016/S0964-8305(01)00036-1

[23] Mildenberger U, Khare A (2000) Planning for an environment-friendly car. Technovation 20(4):205–214. 10.1016/S0166-4972(99)00111-X

[24] Rivera JL, Reyes-Carrillo T (2014) A framework for environmental and energy analysis of the automobile painting process. Procedia Cirp 15:171–175. 10.1016/j.procir.2014.06.022

[25] Aksu Z, Karabayır G (2008) Comparison of biosorption properties of different kinds of fungi for the removal of Gryfalan black RL metal-complex dye. Bioresour Technol 99(16):7730–7741. 10.1016/j.jenvman.2010.02.026 18325761 10.1016/j.biortech.2008.01.056

[26] Di Pippo F, Di Gregorio L, Congestri R, Tandoi V, Rossetti S (2018) Biofilm growth and control in cooling water industrial systems. FEMS Microbiol Ecol 94(5):fiy044. 10.1093/femsec/fiy044 10.1093/femsec/fiy044 29596620

[27] Hickey AM, Gordon L, Dobson AD, Kelly CT, Doyle EM (2007) Effect of surfactants on fluoranthene degradation by *Pseudomonas alcaligenes* PA-10. Appl Microbiol Biotechnol 74:851–856. 10.1007/s00253-006-0719-5 17106676 10.1007/s00253-006-0719-5

[28] Huang WC, Tang IC (2007) Bacterial and yeast cultures–process characteristics, products, and applications. *Bioprocessing for value-added products from renewable resources*, 185–223. 10.1016/B978-044452114-9/50009-8

[29] Jin J, Yao J, Liu W, Zhang Q, Liu J (2017) Fluoranthene degradation and binding mechanism study based on the active-site structure of ring-hydroxylating dioxygenase in *Microbacterium paraoxydans* JPM1. Environ Sci Pollut Res 24:363–371. 10.1007/s11356-016-7809-4 10.1007/s11356-016-7809-4 27722881

[30] Kim KK, Park HY, Park W, Kim IS, Lee ST (2005) *Microbacterium xylanilyticum* sp. nov., a xylan-degrading bacterium isolated from a biofilm. Int J Syst Evol Microbiol 55(5):2075–2079. 10.1099/ijs.0.63706-0 16166712 10.1099/ijs.0.63706-0

[31] Chien CC, Lin BC, Wu CH (2013) Biofilm formation and heavy metal resistance by an environmental *Pseudomonas* sp. Biochem Eng J 78:132–137. 10.1016/j.bej.2013.01.014

[32] Książek-Trela P, Figura D, Węzka D, Szpyrka E (2024) Degradation of a mixture of 13 polycyclic aromatic hydrocarbons by commercial effective microorganisms. Open Life Sci. 10.1515/biol-2022-0831 38415204 10.1515/biol-2022-0831 PMC10898624

[33] Mergaert J, Cnockaert MC, Swings J (2003) *Thermomonas fusca* sp. nov. And *Thermomonas brevis* sp. nov., two mesophilic species isolated from a denitrification reactor with Poly (ε-caprolactone) plastic granules as fixed bed, And emended description of the genus *Thermomonas*. Int J Syst Evol MicroBiol 53(6):1961–1966. 10.1099/ijs.0.02684-0 14657130 10.1099/ijs.0.02684-0

[34] Mergeay M, Van Houdt R (2021) *Cupriavidus metallidurans* CH34, a historical perspective on its discovery, characterization and metal resistance. FEMS Microbiol Ecol 97(2):fiaa247. 10.1093/femsec/fiaa247 33270823 10.1093/femsec/fiaa247

[35] Yu Y, Su J, Xu J, Li YP, Alwathnani HA, Wu Z, Ji C, Feng R, Rensing C, Herzberg M (2022) As (III) exposure induces a zinc scarcity response and restricts iron uptake in High-Level Arsenic-Resistant *Paenibacillus taichungensis* strain NC1. Appl Environ Microbiol 88(9):e00312–e00322. 10.1128/aem.00312-22 35435714 10.1128/aem.00312-22 PMC9088362

[36] Diels L, Van Roy S, Taghavi S, Van Houdt R (2009) From industrial sites to environmental applications with *Cupriavidus metallidurans*. Antonie Van Leeuwenhoek 96(2):247–258. 10.1007/s10482-009-9361-4 19582590 10.1007/s10482-009-9361-4

[37] Grady EN, MacDonald J, Liu L, Richman A, Yuan ZC (2016) Current knowledge and perspectives of *Paenibacillus*: a review. Microb Cell Fact 15(1):203. 10.1186/s12934-016-0603-7 27905924 10.1186/s12934-016-0603-7 PMC5134293

[38] Xia X, Li J, Liao S, Zhou G, Wang H, Li L, Xu B, Wang G (2016) Draft genomic sequence of a chromate-and sulfate-reducing *Alishewanella* strain with the ability to bioremediate Cr and Cd contamination. Stand Genomic Sci 11:1–8. 10.1186/s40793-016-0169-3 27499827 10.1186/s40793-016-0169-3 PMC4974768

[39] Samadi A, Sharifi H, Nejad G, Hasan-Zadeh Z, A., Yaghmaei S (2020) Biodegradation of 4-Chlorobenzoic acid by *Lysinibacillus macrolides* DSM54T and determination of optimal conditions. Int J Environ Res 14:145–154. 10.1007/s41742-020-00247-4

[40] Charles CJ, Rout SP, Jackson BR, Boxall SA, Akbar S, Humphreys PN (2022) The evolution of alkaliphilic biofilm communities in response to extreme alkaline pH values. Microbiol Open 11(4):e1309. 10.1002/mbo3.1309 10.1002/mbo3.1309 PMC9380404 36031955

[41] Lee YS, Park W (2019) Enhanced calcium carbonate-biofilm complex formation by alkali-generating *Lysinibacillus boronitolerans* YS11 and alkaliphilic *Bacillus* sp. AK13. Amb Express 9(1):49. 10.1186/s13568-019-0773-x 30976947 10.1186/s13568-019-0773-x PMC6459448

[42] Tay CC, Mohamad-Nasir N, Hashim SN, Lokman NF, Wong KK (2024) Different enzymatic strategy to degrade carbamazepine by *Rhodococcus zopfii* and *Leifsonia shinshuensis*. Proc Natl Acad Sci India Sect B Biol Sci. 10.1007/s40011-023-01539-3

[43] Méndez V, Holland S, Bhardwaj S, McDonald J, Khan S, O’Carroll D, Pickford R, Richards S, O’Farrell C, Coleman N, Lee M, Manefield MJ (2022) Aerobic biotransformation of 6: 2 fluorotelomer sulfonate by *Dietzia aurantiaca* J3 under sulfur-limiting conditions. Sci Total Environ 829:154587. 10.1016/j.scitotenv.2022.154587 35306084 10.1016/j.scitotenv.2022.154587

[44] Ramya Devi KC, Sundaram RL, Vajiravelu S, Vasudevan V, Mary Elizabeth GK (2019) Structure elucidation and proposed de novo synthesis of an unusual mono-rhamnolipid by *Pseudomonas guguanensis* from Chennai Port area. Sci Rep 9(1):5992. 10.1038/s41598-019-42045-9 30979908 10.1038/s41598-019-42045-9 PMC6461634

[45] Oztas Gulmus E, Gormez A (2020) Characterization and biotechnological application of protease from thermophilic *Thermomonas haemolytica*. Arch Microbiol 202(1):153–159. 10.1007/s00203-019-01728-7 31541265 10.1007/s00203-019-01728-7

[46] Khordadmehr M, Ghaderi S, Mesgari-Abbasi M, Jigari-Asl F, Nofouzi K, Tayefi-Nasrabadi H, McIntyre G (2021) The beneficial effects of actinomycetales immune modulators in the pancreas of diabetic rats. Adv Pharm Bull 11(2):371. 10.34172/apb.2021.035 33880360 10.34172/apb.2021.035 PMC8046390

[47] Biedendieck R, Knuuti T, Moore SJ, Jahn D (2021) The beauty in the beast—the multiple uses of *Priestia megaterium* in biotechnology. Appl Microbiol Biotechnol 105:5719–5737. 10.1007/s00253-021-11424-6 34263356 10.1007/s00253-021-11424-6 PMC8390425

[48] Mendo S, Faustino NA, Sarmento AC, Amado F, Moir AJ (2004) Purification and characterization of a new peptide antibiotic produced by a thermotolerant *Bacillus licheniformis* strain. Biotechnol Lett 26:115–119. 10.1023/B:BILE.0000012888.72489.3f 15000477 10.1023/b:bile.0000012888.72489.3f

[49] Su Y, Liu C, Fang H, Zhang D (2020) *Bacillus subtilis*: a universal cell factory for industry, agriculture, biomaterials and medicine. Microb Cell Fact 19:1–12. 10.1186/s12934-020-01436-8 32883293 10.1186/s12934-020-01436-8 PMC7650271

[50] Partovinia A, Soorki AA, Koosha M (2021) Synergistic adsorption and biodegradation of heavy crude oil by a novel hybrid matrix containing immobilized *Bacillus licheniformis*: aqueous phase and soil bioremediation. Ecotoxicol Environ Saf 222:112505. 10.1016/j.ecoenv.2021.112505 34273849 10.1016/j.ecoenv.2021.112505

[51] Romo-Barrera CM, Castrillón-Rivera LE, Palma-Ramos A, Castañeda-Sánchez JI, Luna-Herrera J (2021) *Bacillus licheniformis* and *Bacillus subtilis*, probiotics that induce the formation of macrophage extracellular traps. Microorganisms 9(10):2027. 10.3390/microorganisms9102027 34683348 10.3390/microorganisms9102027 PMC8540962

[52] de Nunes O, Medeiros PS, De FH, de Oliveira TS, de Almeida Zago JR, Bettiol W (2023) Bacillus subtilis and Bacillus licheniformis promote tomato growth. Brazilian J Microbiol 54(1):397–406. 10.1007/s42770-022-00874-3 10.1007/s42770-022-00874-3 PMC9943921 36422850

[53] Shemesh M, Ostrov I (2020) Role of *Bacillus* species in biofilm persistence and emerging antibiofilm strategies in the dairy industry. J Sci Food Agric 100(6):2327–2336. 10.1002/jsfa.10285 31975392 10.1002/jsfa.10285

[54] Mechichi T, Stackebrandt E, Fuchs G (2003) *Alicycliphilus denitrificans* gen. nov., sp. nov., a cyclohexanol-degrading, nitrate-reducing β-proteobacterium. Int J Syst Evol Microbiol 53(1):147–152. 10.1099/ijs.0.02276-0 12661531 10.1099/ijs.0.02276-0

[55] Song J, Dong L, Ding Y, Zhou J (2021) A case report of brain abscess caused by *Nocardia farcinica*. Eur J Med Res 26:1–7. 10.1186/s40001-021-00562-2 34344465 10.1186/s40001-021-00562-2 PMC8330121

[56] Jovanovic J, Ornelis VF, Madder A, Rajkovic A (2021) *Bacillus cereus* food intoxication and toxicoinfection. Compr Rev Food Sci Food Saf 20(4):3719–3761. 10.1111/1541-4337.12785 34160120 10.1111/1541-4337.12785

[57] Brown MM, Horswill AR (2020) *Staphylococcus epidermidis*—skin friend or foe? PLoS Pathog 16(11):e1009026. 10.1371/journal.ppat.1009026 33180890 10.1371/journal.ppat.1009026 PMC7660545

[58] Lawal OU, Fraqueza MJ, Bouchami O, Worning P, Bartels MD, Gonçalves ML, Paixão P, Gonçalves E, Toscano C, Empel J, Urbas M, Domínguez MA, West H, de Lencastre H, Miragaia M (2021) Foodborne origin and local and global spread of *Staphylococcus saprophyticus* causing human urinary tract infections. Emerg Infect Dis 27(3):880. 10.3201/eid2703.200852 33622483 10.3201/eid2703.200852 PMC7920669

[59] Heath V, Cloutman-Green E, Watkin S, Karlikowska M, Ready D, Hatcher J, Pearce-Smith N, Brown C, Demirjian A (2023) *Staphylococcus capitis*: review of its role in infections and outbreaks. Antibiotics 12(4):669. 10.3390/antibiotics12040669 37107031 10.3390/antibiotics12040669 PMC10135222

[60] Miltiadous G, Elisaf M (2011) Native valve endocarditis due to *Micrococcus luteus*: a case report and review of the literature. J Med Case Rep 5:1–3. 10.1186/1752-1947-5-251 21714882 10.1186/1752-1947-5-251 PMC3141704

[61] Sakuma M, Hashimoto M, Nishi K, Tohya M, Hishinuma T, Shimojima M, Tada T, Kirikae T (2023) Emergence of colistin-resistant *Acinetobacter modestus* harbouring the intrinsic phosphoethanolamine transferase EptA. J Glob Antimicrob Resist 33:101–108. 10.1016/j.jgar.2023.02.023 36906175 10.1016/j.jgar.2023.02.023

[62] Aguilar-Vera A, Bello-López E, Pantoja-Nuñez GI, Rodríguez-López GM, Morales-Erasto V, Castillo-Ramírez S (2024) *Acinetobacter junii*: an emerging one health pathogen. Msphere e00162–e00124. 10.1128/msphere.00162-24 10.1128/msphere.00162-24 PMC11237400 38606973

[63] Martí S, Rodríguez-Baño J, Catel-Ferreira M, Jouenne T, Vila J, Seifert H, Dé E (2011) Biofilm formation at the solid-liquid and air-liquid interfaces by *Acinetobacter* species. BMC Res Notes 4(1):5. 10.1186/1756-0500-4-5 21223561 10.1186/1756-0500-4-5 PMC3023692

[64] Tarifa MC, Lozano JE, Brugnoni LI (2018) Disinfection efficacy over yeast biofilms of juice processing industries. Food Res Int 105:473–481. 10.1016/j.foodres.2017.11.018 29433238 10.1016/j.foodres.2017.11.018

[65] Bertini A, De Bernardis F, Hensgens LA, Sandini S, Senesi S, Tavanti A (2013) Comparison of *Candida parapsilosis*, *Candida orthopsilosis*, and *Candida metapsilosis* adhesive properties and pathogenicity. Int J Med Microbiol 303(2):98–103. 10.1016/j.ijmm.2012.12.006 23403338 10.1016/j.ijmm.2012.12.006

[66] Pál SE, Tóth R, Nosanchuk JD, Vágvölgyi C, Németh T, Gácser A (2021) A *Candida parapsilosis* overexpression collection reveals genes required for pathogenesis. J Fungi 7(2):97. 10.3390/jof7020097 10.3390/jof7020097 PMC7911391 33572958

[67] Kovaleva J, Degener JE, Van der Mei HC (2010) Mimicking disinfection and drying of biofilms in contaminated endoscopes. J Hosp Infect 76(4):345–350. 10.1016/j.jhin.2010.07.008 20951470 10.1016/j.jhin.2010.07.008

[68] Pierantoni DC, Bernardo M, Mallardo E, Carannante N, Attanasio V, Corte L, Roscini L, Di Fiore L, Tascini C, Cardinali G (2020) *Candida palmioleophila* isolation in Italy from two cases of systemic infection, after a CHROMagar and Vitek system mis-identification as *C. albicans*. New Microbiol 43(1):47–50 31814032

[69] Wu N, Wu Y, Chu Y, Ren Z, Li H, Rong C, Yang M, Jiang N, Jiang Y, Chen J, Zhang J, Tian S (2023) The first rare case of *Candida palmioleophila* infection reported in China and its genomic evolution in a human host environment. Front Microbiol. 10.3389/fmicb.2023.1165721 37664129 10.3389/fmicb.2023.1165721 PMC10469324

[70] Campos JM, Stamford TLM, Sarubbo LA (2019) Characterization and application of a biosurfactant isolated from *Candida utilis* in salad dressings. Biodegradation 30:313–324. 10.1007/s10532-019-09877-8 31089840 10.1007/s10532-019-09877-8

[71] Khanna A, Handa S, Rana S, Suttee A, Puri S, Chatterjee M (2023) Biosurfactant from *Candida*: sources, classification, and emerging applications. Arch Microbiol 205(4):149. 10.1007/s00203-023-03495-y 36995448 10.1007/s00203-023-03495-y

[72] Zhao Y, Song B, Li J, Zhang J (2022) *Rhodotorula toruloides*: an ideal microbial cell factory to produce oleochemicals, carotenoids, and other products. World J Microbiol Biotechnol 38:1–19. 10.1007/s11274-021-03201-4 10.1007/s11274-021-03201-4 34873661

